# The Value of Early and Follow-Up Elevated Scores Based on Peripheral Complete Blood Cell Count for Predicting Adverse Outcomes in COVID-19 Patients

**DOI:** 10.3390/jpm12122037

**Published:** 2022-12-09

**Authors:** Andrei-Costin Chelariu, Adorata Elena Coman, Catalina Lionte, Victoria Gorciac, Victorita Sorodoc, Raluca Ecaterina Haliga, Ovidiu Rusalim Petris, Cristina Bologa, Gabriela Puha, Alexandra Stoica, Mihai Constantin, Oana Sirbu, Alexandr Ceasovschih, Laurentiu Sorodoc

**Affiliations:** 1 Hematology Department, Regional Institute of Oncology, 700483 Iasi, Romania; 2 Second Internal Medicine Clinic, “Sf. Spiridon” Emergency Clinical County Hospital, 700111 Iasi, Romania; 3 Preventive Medicine and Interdisciplinarity Department, “Grigore T. Popa” University of Medicine and Pharmacy Iasi, 700115 Iasi, Romania; 4 Internal Medicine Department, “Grigore T. Popa” University of Medicine and Pharmacy Iasi, 700115 Iasi, Romania; 5 Rheumatology Department, Clinical Rehabilitation Hospital, 700661 Iasi, Romania; 6 Nursing Department, “Grigore T. Popa” University of Medicine and Pharmacy Iasi, 700115 Iasi, Romania

**Keywords:** critical care, short-term mortality, neutrophil-to-lymphocyte ratio, monocyte-to-lymphocyte ratio, COVID-19, outcome

## Abstract

Background: The ongoing COVID-19 pandemic has put a constant strain on hospital resources, so there is a dire need for investigation methods that are widely available and that can predict mortality and the need for critical care. Hematological indices, which can be easily calculated from a complete blood count (CBC), are useful in determining a patient’s inflammatory response to infectious diseases. Aim: This was a prospective cohort study that aimed to assess the prognostic value of scores based on CBCs in hospitalized patients with mild or moderate COVID-19 and medical comorbidities regarding the need for intensive care unit (ICU) therapy and short-term mortality. Methods: We included 607 patients with confirmed COVID-19, followed up for the need for ICU admission (15.5%) and 30 day mortality post-discharge (21.7%). CBC-derived scores were tested upon emergency department (ED) admission and after a median of 8 days. Results: In a multivariate model, elevated followed-up neutrophil-to-lymphocyte ratio (NLR) predicted increased odds for ICU admission (OR: 1.14 [95%CI: 1.06–1.22], *p* < 0.001) and short-term mortality (OR: 1.30 [95%CI: 1.09–1.57], *p* = 0.005). Monocyte-to-lymphocyte ratio (MLR) predicted 2.5-fold increased odds for ICU admission and 2.2-fold increased odds for mortality. Conclusion: NLR and MLR followed up 8 days post-admission are predictive for adverse outcomes in mild or moderate COVID-19 patients.

## 1. Introduction

The coronavirus disease has affected the entire world, with more than 622 million confirmed cases and 6.5 million deaths according to World Health Organization current data [1]. This has undoubtedly placed a large burden on the population, health system and economy of every country. Thus, there is a need for cheaper laboratory investigations that can accurately determine disease progression towards negative outcomes.

Circulating biomarkers of inflammation (i.e., C-reactive protein, neutrophils, eosinophils, basophils and monocytes) and inflammatory scores derived from the peripheral complete blood count (CBC), such as the neutrophil-to-lymphocyte ratio (NLR), monocyte-to-lymphocyte ratio (MLR) and platelet-to-lymphocyte ratio (PLR), are quick, easy-to-obtain, inexpensive and widely available hematological indices that give insight into a patient’s inflammatory response to certain illnesses spanning multiple fields of medicine. Most commonly, these hematological indices have been used in oncology, where elevated values for the NLR and PLR can be predictors of poor outcomes in patients with non-small cell lung cancers [2], gastrointestinal cancers [3,4] and breast cancer [5], as well as several other malignancies. Other applications relate to myocardial diseases and cardiogenic shock [6,7,8]; infectious diseases, as predictors of sepsis and bacteremia [9,10]; and acute poisoning, as predictors for ICU admission and mortality [11].

Virus infection can cause a variety of hematological changes. The degree of the changes in leukocytes, neutrophils, lymphocytes, monocytes, eosinophils, basophils, platelets, hemoglobin levels, mean corpuscular volume (MCV) and mean cell hemoglobin concentration (MCHC) are overall associated with lung involvement, oxygen demand and disease activity in COVID-19 patients. C-reactive protein (CRP), lactate dehydrogenase (LDH), the platelet-to-lymphocyte ratio (PLR) and the NLR are significantly higher in COVID-19 patients compared to patients who do not have COVID-19 [12]. Marked elevations in hematologic biomarkers, such as LDH, D-dimer, ferritin and CRP, are associated with worse outcomes in COVID-19 [13]. Moreover, lymphocytopenia, NLR and platelets were identified as risk factors for survival in patients with COVID-19 [14]. High NLR concentrations enhance the symptoms’ severity and, thus, the mortality rate in COVID-19 [15].

Regarding COVID-19, several studies have analyzed the use of the NLR, MLR and PLR, among various other markers, as predictors of disease severity and the need for intensive care and mortality. Unlike our study, they mostly focused, with regard to intensive care unit (ICU) admission and in-hospital mortality, on patients with severe COVID-19 and generally used cohorts of fewer patients. The C-reactive protein/lymphocyte ratio (CLR) combined with the NLR was found to be predictive for mortality in COVID-19 patients with refractory disease admitted to the ICU [16]. A recent meta-analysis showed that severe COVID-19 patients and non-survivors of COVID-19 had higher NLR levels upon admission than non-severe cases and survivors. However, to date, no optimal cut-off value has been validated across different populations [17]. As vaccination results in the activation of T and B lymphocytes, which could last for months, the NLR may not be a useful laboratory marker in vaccinated patients with COVID-19 [18].

Our study aimed to identify whether CBC-derived scores determined early, upon admission to the emergency department (ED), were useful for the prediction of the evolution towards a severe disease and the need for critical care in a large cohort of patients with non-critical COVID-19 disease and medical comorbidities. We also studied the evolution of these indices after a follow up of 8 (range 7–10) days and their relationship with ICU admission and short-term mortality. To our knowledge, no other studies have analyzed these indices with follow up. If these parameters can serve as predictors of disease severity, considered as the need for intensive care, then we have another inexpensive and widely available tool to identify severe cases of infection.

## 2. Materials and Methods

This study was undertaken in line with the Strengthening the Reporting of Observational Studies in Epidemiology (STROBE) guidelines for observational studies.

This was a prospective cohort study aimed at assessing the prognostic value of hematological indices in hospitalized patients with mild or moderate COVID-19 regarding the evolution towards critical disease and the need for ICU therapy. Patients aged over 18 years who were admitted for a medical condition to the Internal Medicine Department of “Sf. Spiridon” Emergency County Hospital in Iasi, Romania, between 1 October 2020 and 30 April 2022 and had confirmation of SARS-CoV-2 infection from an RNA reverse-transcriptase polymerase chain reaction assay were included in this study. Patients under 18, discharged against medical advice or with incomplete data upon admission to ED or follow up were excluded, while patients with multiple readmissions during the study period were evaluated as a single presentation. The patients were followed up 30 days post-discharge to evaluate short-term mortality. This study was approved by our Institutional Review Board, and individual written informed consent was waived based on legal standards for national healthcare alarm situations.

For data extraction, we recorded demographics, vital signs, body mass index (BMI) and comorbidities. The studied variables were: neutrophil-to-lymphocyte ratio (NLR), platelet-to-lymphocyte ration (PLR), monocyte-to-lymphocyte ratio (MLR) and systemic inflammation index (SII), as well as neutrophil-to-monocyte ratio (NMR) and neutrophil-to-platelet ratio (NPR), erythrocyte sedimentation rate (ESR), red cell distribution width (RDW), mean platelet volume (MPV), mean platelet volume (MPV) to platelet count (PC) ratio (MPR), hemoglobin, white blood cells (WBCs), prothrombin time (PT), fibrinogen, C-reactive protein (CRP), C-reactive protein-to-lymphocyte ratio (CLR), creatinine, TGP, lactate dehydrogenase (LDH), presepsin and ferritin. The hematological indices were calculated using the mathematical division of their absolute values derived from CBCs from peripheral blood samples available upon ED admission and after follow up of a median 8 days (range 7–10). Additionally, the National Early Warning Score (NEWS2) [19] was extracted from clinical records and the Charlson comorbidity index (CCI) was calculated according to the scoring system established by Charlson et al. [20]. Biochemistry and hematology results upon admission and follow up, in-hospital clinical course, in-hospital complications, treatment and outcomes were extracted from each patient’s electronic medical record. A PATHFAST Cardiac Biomarker Analyzer (LSI Medience Corporation, Tokyo, Japan), Sysmex XT-4000i—Automated Hematology Analyzer (Sysmex Corporation, Tokyo, Japan) and ARCHITECT c16000 clinical chemistry analyzer (Abbott Laboratories, Chicago, IL, USA) were used for testing hematology and biochemistry samples.

Categorical variables were summarized as percentages and continuous variables as the number of non-missing observations and the mean and standard deviation (SD) or median and interquartile range (IQR). Variables were analyzed using Mann–Whitney or Chi-squared tests as appropriate. Missing data were excluded listwise where applicable. To evaluate independent factors, univariate and multivariate logistic regressions (enter method) were performed. Significant variables with a *p* value < 0.1 were included in the multivariate models (CCI and NEWS2 score; viral strain; comorbidities, including oncologic diseases; and the inflammation biomarkers previously associated with the outcomes in COVID-19 patients). Age was excluded from the list of potential confounders because it is already included in CCI score and is one of the criteria for giving patients access to ICU care. The calibration of the models was assessed using the Hosmer–Lemeshow goodness-of-fit statistic (good fit was defined as a *p* value of >0.05). We used bootstrapping for internal validation of the models. Data are presented as odds ratios (ORs) and 95% confidence intervals (CIs). Receiver operating characteristic curves (ROCs) were built to analyze the diagnostic performance of the multivariate models for prediction of admission to the ICU and 30 day mortality. In addition, we calculated the positive predictive value (PPV) and the negative predictive value (NPV) of the models applied. A statistical test was significant when the *p* value was <0.05. All *p* values were the results of two-tailed tests. Statistical analyses were performed using SPSS software version 22 (IBM Corp, Chicago, IL, USA).

## 3. Results

**Figure 1:**
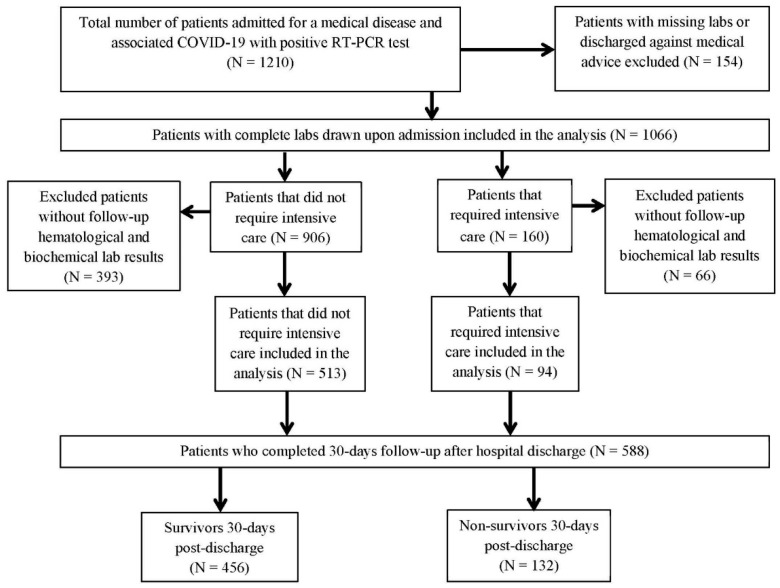
Flow chart of the study cohort.

We included 607 hospitalized patients (median age of 70 years, 294 males (48.4%)) with a medical disease and associated confirmed mild or moderate COVID-19 as defined according to CDC guidelines [21]. The main reasons for patients’ admission were: ischemic heart disease (22.8%), dysrhythmias (25.4%), decompensated heart failure (16.5%), acute decompensation of chronic kidney disease (20.6%), exacerbation of a chronic pulmonary disease (8.3%), decompensated liver cirrhosis (4.4%) and dyselectrolytemia (2%). We recorded 94 patients (15.5%) who developed severe forms of COVID-19 and required transfer to the ICU (Figure 1).

The viral strains involved were the Alpha variant in 362 patients (59.6%), the Delta variant in 129 patients (21.3%), the Beta variant in 64 cases (10.5%) and Omicron in 52 cases (8.6%). We observed that viral strain significantly influenced the main outcomes analyzed. The patients infected with the Delta variant had significantly higher rates of ICU admission (52.1% vs. 15.6%, *p* < 0.001). Moreover, 30 day survival was significantly influenced by the viral strain, the Alpha variant and the Delta variant being responsible for 47.7% and 25% of deaths (*p* < 0.001), respectively. The oncologic comorbidities associated were solid cancers in 12 patients (2%) and hematologic malignancies in 102 patients (16.8%). The presence of an oncologic disease (OD), defined as a hematologic malignancy or a solid cancer, significantly influenced 30 day mortality (15% vs. 9.8%, *p* = 0.045). The median number of days in hospital was 12 days for patients who did not need transfer to the ICU and 10 days for patients transferred to the ICU. Thirty days after hospital discharge, data were available for 588 patients. Baseline characteristics of the cohort according to the main outcomes are presented in Table 1. The mortality rate 30 days post-discharge was 22.44%. Given the small number of vaccinated patients (11.9%), the vaccination status could not have significantly influenced the outcomes. However, we observed a tendency towards lower rates for ICU admission in vaccinated patients, but without statistical significance (12% vs. 88%, *p* = 0.072). 

We tested CBC and CBC-derived parameters, such as RDW, NLR, MLR, PLR, SII, CRP and CLR, as well as other inflammation-related markers (i.e., fibrinogen and LDH), upon admission and follow up. Other CBC-derived scores, such as NMR and NPR, were available only upon admission, as was the case for ESR, arterial lactate, presepsin, and ferritin. Lymphocytopenia, defined as lymphocyte count <1.5 × 10^3^/mmc, was present upon admission in 71.2% cases and on follow up in 57.3% patients. We noticed that patients who evolved towards a critical disease had significantly higher WBC values upon admission and follow up and significantly lower lymphocyte counts early after admission and after 8 days follow up. Furthermore, arterial lactate upon admission was significantly increased. Only follow-up WBCs and lymphocyte counts were significantly correlated with 30 day mortality. However, these variables had no significant influence on the outcomes in the logistic regression analysis.

### 3.1. CBC-Derived Scores in Relation to ICU Admission

**Figure 2:**
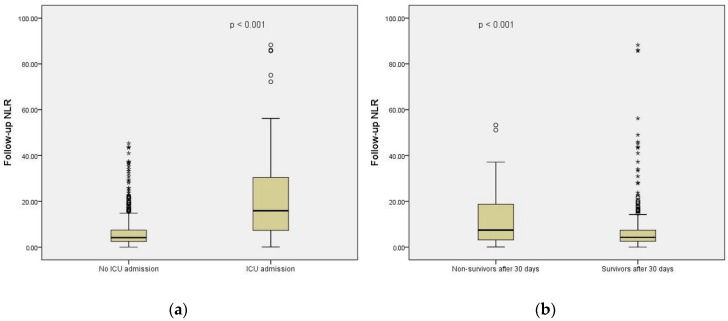
Boxplots displaying NLR values according to the main outcomes: (**a**) follow-up NLR was significantly higher in patients admitted to ICU; (**b**) follow-up NLR was significantly higher in non-survivors 30 days post-discharge; ° represent outliers; * represent extreme values.

We noticed that admission CBC-derived indexes, apart from RDW, MPV and MPR, were significantly higher in patients who needed critical care, while at follow up, only the NLR, MLR, PLR, SII and CLR had significantly higher values in patients with need of ICU therapy. The PLR, NMR, NPR and CLR were found to be statistically significant in the univariate analysis (*p* < 0.05); however, in multivariate models, they were not significant or had little influence on the model itself. The NLR upon admission to the ED was statistically significant for our established outcome; however, we could not obtain a model with satisfactory sensitivity and specificity in the ROC curve. The follow-up values for the NLR (Figure 2a) and MLR were higher in patients who developed severe disease, required ICU admission and had higher odds ratios for predicting ICU admission in the multivariate models. These indices were tested both independently and in a combined model. 

**Figure 3:**
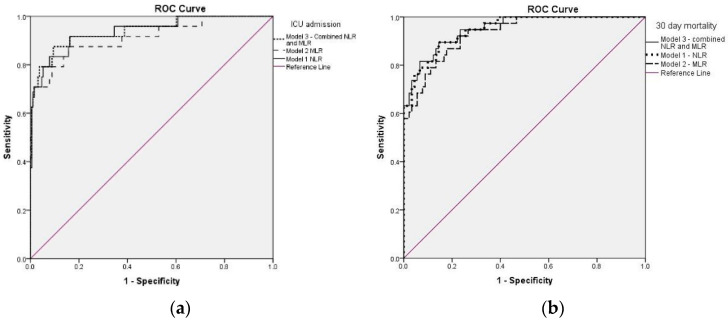
ROC curve for the predictive models including NLR and MLR in relation to the main outcomes: (**a**) the predictive models for ICU admission; (**b**) the predictive models for short-term mortality.

The NLR on follow up was tested through multivariate logistical regression alongside other inflammatory response markers, such as fibrinogen, CRP, LDH, presepsin and history of OD. In model 1, SII and presepsin showed no influence on the established outcome, while follow-up NLR significantly predicted 14% increased risk for ICU admission (Table 2). The viral strain Delta predicted more than twofold increased odds for ICU therapy. The resulting ROC (Figure 3a) had an area under the curve (AUC) of 0.937 (95%CI: 0.880–0.994, *p* < 0.001). The PPV of the model was 98%, and the NPV was 31%.

Follow-up MLR was tested using a multivariate model including significant covariates (model 2). MLR predicted 2.4-fold increased odds for ICU admission and the viral strain Delta showed more than twofold increased odds for ICU admission, while SII, presepsin and LDH showed no influence for this outcome. Notably, SaO2 upon admission (<90%) predicted fivefold increased odds for the need for ICU therapy (Table 3). The resulting ROC curve (Figure 3a) had an AUC of 0.912 (95%CI: 0.838–0.986, *p* < 0.001), and the PPV was 97% and the NPV was 34%.

Both the follow-up NLR and MLR were tested in a combined model using the same variables as the independent models for ICU admission (model 3). Out of the two, only follow-up NLR remained a statistically significant predictor for this outcome (Supplementary Table S1). Follow-up MLR, despite having a good predictive accuracy, failed to obtain statistical significance. The ROC from this model had an AUC of 0.939 (95%CI: 0.882–0.997, *p* < 0.001) with a PPV of 98% and an NPV of 36%. Therefore, there is little benefit from a combined model when compared to independent models regarding ICU admission. 

### 3.2. CBC-Derived Scores in Relation to Short-Term Mortality

Follow-up hematological indices, including NLR, MLR, PLR and SII, and inflammatory biomarkers, including CRP, CLR, fibrinogen and LDH, assessed pre-discharge were significantly increased in non-survivors. However, only NLR, MLR, CCI and LDH showed significant predictive values for short-term mortality in our cohort. Further, the duration of illness significantly influenced this outcome. Follow-up NLR predicted a 30% increase in short-term mortality in model 1, which included pre-discharge CRP, fibrinogen, LDH SII, the duration of illness and the presence of an OD (Table 2). The resulting ROC curve (Figure 3a) had an AUC of 0.948 (95%CI 0.91–0.984, *p* < 0.001). The PPV of this model was 93% and the NPV 72%.

Although admission MLR was significantly increased in patients who did not survive 30 days post-discharge, in the multivariate analysis, only follow-up MLR significantly influenced short-term mortality. In model 2, which included pre-discharge CRP and LDH, fibrinogen, SII, CCI score, duration of sickness, the presence of an OD and admission SaO2 (Table 3), follow-up MLR predicted a 2.2-fold increase in short-term mortality. The resulting ROC curve (Figure 3b) had an AUC of 0.931 (95%CI: 0.888–0.975, *p* < 0.001). The PPV of the model was 90%, and the NPV was 52%.

The multivariate analysis regarding short-term mortality showed the highest AUC of 0.950 (95%CI: 0.914–0.986, *p* < 0.001) for the combined model including follow-up NLR and MLR (Figure 3b). However, in the combined model (model 3), only follow-up NLR remained statistically significant for this outcome (Table 4, Supplementary Table S1), and the PPV and NPV were not different from model 1. Therefore, it is important to assess either follow-up NLR or follow-up MLR to predict short-term mortality.

## 4. Discussion

This study provides new data regarding the use of CBC-derived scores upon ED admission, as well as after a median follow up of 8 days, in hospitalized patients with medical comorbidities and mild or moderate COVID-19 in relation to the need for critical care and short-term mortality post-discharge. Firstly, we assessed a higher number of CBC-derived scores as predictors for ICU admission and short-term mortality than previously published studies [12,14,16,22,23,24,25]. Secondly, and for the first time, we assessed the value of followed-up CBC-derived scores in relation to these outcomes. In our cohort, patients with medical diseases and associated non-critical COVID-19 with need of ICU admission and short-term mortality had higher NLR values obtained upon admission and on follow up compared to the values above 5.92 reported in previous studies for COVID-19 patients with higher rates of ICU admission and mortality [22]. We recorded a low number of patients with histories of hematologic malignancies or solid cancers to quantify the exact influence of these comorbidities on the outcomes. However, in our cohort, patients had a higher NLR value upon admission compared to the value of 3.46 reported in a study that analyzed patients with hematological cancers and COVID-19. Moreover, in the aforementioned study, the type of hematological malignancy failed to predict the prognosis of COVID-19 after multivariate analysis [26].

Elevated values for the NLR, MLR and PLR are likely to be the result of lymphopenia, which develops during COVID-19 infection. Indeed, we recorded lymphopenia in a significant percentage of our patients upon admission, as well as after 8 days follow up, which was in accordance with other studies that reported that lymphocytopenia drops to its nadir 8 days after admission [14]. Studies have shown that lymphopenia occurs due to the decline in CD4+ and CD8+ T cells caused by multiple factors. As lymphocytes are dominant in the interstitial area of the lung, the increased immune activation, elevated lung cytokine/chemokine levels and consequent lung injury present in COVID-19 infection explain the lymphopenia [21,23]. Additionally, most COVID-19 patients also develop neutrophilia and thrombocytopenia over the course of the infection [12], which could explain the higher impact of increased NLR on severity and mortality. Although some studies have found a correlation between elevated PLR and disease severity [27], combined thrombocytopenia and lymphopenia would result in smaller changes in PLR values overall and would not serve as a good predictor of disease and mortality, which concurs with our findings.

Several studies have tried to determine the feasibility of using hematological indices to predict severity of disease and mortality in COVID-19 patients alongside other markers, such as CRP and ferritin. Yildirim et al. studied the NLR, NPR, NMR and CRP in 160 patients with COVID-19 pneumonia and found them to be good predictors of mortality and intensive care need if the values were above a cut-off of 2.9 for NLR and 19.7 for CRP; however, CRP had better specificity and sensitivity in the ROC analysis [24]. In our population, CRP did not improve the accuracy of the model. This can be explained by the fact that, in the aforementioned study, the patients had moderate to severe forms of disease, while in our cohort, we included patients with mild or moderate COVID-19. Agarwal R. et al. studied 468 patients with COVID-19 infection and other comorbidities, such as diabetes, chronic kidney disease and heart failure. They found that patients with NLR values higher than the 4.42 cut-off presented with more comorbidities and had echocardiographic changes, such as left or right ventricle dysfunction or valve abnormalities [28]. Our results showed that, in a cohort of patients with complex medical diseases and mild or moderate COVID-19, NLR upon admission was increased in all patients more than the cut-off of 4.6 and was significantly higher in those who developed severe disease and needed ICU care. These results are in accordance with the cut-off NLR of ≥4.5 reported by Li et al. for severity [29]. Moreover, the admission MLR was significantly increased in patients with the need for ICU therapy. 

Although Qu et al. found, in a retrospective analysis of 30 COVID-19 patients, that the PLR, a marker of cytokine storms, is associated with prognosis [25], we could not confirm these results in our cohort after multivariate analysis. Another study analyzed the NLR, PLR, lymphocyte-to-monocyte ratio (LMR) and lymphocyte-to-C-reactive protein ratio (LCR) in 304 patients with severe and non-severe disease (ambulatory patients). They showed that the NLR and PLR were higher in patients with severe clinical symptoms, while the LCR was lower and the LMR was not statistically significant [30]. In our study, we used the CLR instead of the LCR and the MLR instead of the LMR. After multivariate analysis, we observed a significant predictive value for the evolution to a critical disease and need for ICU admission only for the MLR. 

All hematological indices were predictive for ICU therapy both upon admission and on follow up, with the NLR being a better predictor than MLR, while the PLR, SII, NMR and NPR had minimal impacts on this outcome. However, the values of the NLR and MLR on follow up were better predictors for the need for critical care than the same parameters calculated upon admission. This can be explained by the fact that neutrophilia, lymphopenia and thrombocytopenia may develop at different rates over the course of an infection; therefore, patients with mild cases of COVID-19 may present with only one or none of these, whereas patients whose infection progresses towards increased severity may develop all three over a period of time. As a result, NLR and MLR values at a median follow up of 8 days after admission will be higher in patients with more severe forms of COVID-19 due to associated lymphopenia.

In a retrospective cohort study, the NLR, dNLR and MLR determined at hospital admission had high value in predicting in-hospital death among patients with COVID-19 [31]. Zhang et al. identified an NLR ≥ 8 as being associated with 9.7-fold increased odds of 28 day mortality in a univariable Cox regression model of 516 COVID-19 patients [32]. Moreover, Li et al. have reported a cut-off NLR ≥ 6.5 for mortality [29]. In our cohort, both the admission NLR and follow-up NLR were higher than the cut-off identified by these authors. However, this hematological index only predicted a 10% increase in short-term mortality. There is no consensus regarding the optimal NLR cut-off value for determining the elevated level, particularly for COVID-19 patients, since the NLR has been found to vary based on ethnicity, age and sex [33,34]. 

An MLR above 0.69 was associated with 3.29 increased odds of in-hospital mortality in a model adjusted for age, comorbidities, COVID-19 severity and sex [31]. In our study, both admission MLR and follow-up MLR were above this cut-off value in non-survivors. We observed that follow-up MLR was a better predictor of short-term mortality using a model adjusted for clinical characteristics, comorbidities and inflammatory biomarkers.

The CCI has been proven to be a useful tool to prognosticate 30 day mortality, as well as long-term mortality (1 year), in patients with bloodstream infection in the ED [35]. A CCI score ≥ 3 was prognostically associated with mortality in hospitalized COVID-19 patients [36]. Moreover, a CCI ≥ 7 and an NLR ≥ 9 were among the main prognostic indicators for in-hospital mortality in patients with COVID-19 during the Omicron period [37]. We likewise observed a correlation between the NLR and CCI in hospitalized COVID-19 patients with associated medical comorbidities. Moreover, in our regression model for short-term mortality, the NLR and CCI were the strongest predictors. 

The major strengths of our study were the larger sample size compared to other similar studies, including patients with complex medical comorbidities, and a larger timeframe, which made it possible to include many viral strains of concern. We tested more CBC-derived scores than previous studies, and we identified the follow-up NLR and MLR—easy, inexpensive and available markers—as suitable for predicting the need for ICU admission and short-term mortality in a cohort of hospitalized patients with mild or moderate COVID-19. 

One limitation of this study was that data were obtained from a single center in northeastern Romania and may not reflect the entire European population. Another limitation was the lack of ethnic variety in our cohort. A third limitation was the low number of patients with histories of hematologic malignancies or solid cancers, which made it difficult to quantify the exact influence of these comorbidities on the outcomes. A fourth limitation was that not all CBC-derived scores were available on follow up for analysis, and each patient could have been at a different stage of the disease. Finally, the small number of vaccinated patients in our cohort could have impacted the outcomes. 

## 5. Conclusions

Both the NLR and MLR have high value in predicting the need for intensive care and short-term mortality in hospitalized mild or moderate COVID-19 patients with associated medical comorbidities, and their predictive value is increased if they are calculated from a CBC taken 8 days from admission. Although it is arguable whether they alone are enough to determine if a patient needs to be admitted to the ICU, since they are calculated from a CBC, which is a laboratory test that is readily available in almost all hospitals worldwide, they can be used as another tool in the physicians’ arsenal in the fight against the coronavirus pandemic. We recommend systematically following up these indices in non-critically ill COVID-19 patients so that medical professionals can allocate timely ICU resources to the patients in need. Considering the high risk of short-term mortality, elevated follow-up NLR and MLR are significant in terms of planning the necessary strategies to reduce mortality.

## Figures and Tables

**Table 1:** Baseline characteristics of the cohort according to the main outcomes.

Variable	Total Cases N = 607	No ICU Admission N = 513	ICU Admission N = 94	*p* Value	30 Day Survivors N = 456	30 Day Non-Survivors N = 132	*p* Value
Age groups (N, %)				0.949 ^a^			0.002 ^a^
51–60 y	51 (8.4)	43 (8.4)	8 (8.5)		48 (10.5)	5 (3.8)	
61–70 y	141 (23.2)	118 (23.0)	23 (24.5)		115 (25.2)	21 (15.9)	
>70 y	415 (68.4)	352 (68.6)	63 (67.0)		293 (64.3)	106 (80.3)	
Males (N, %)	294 (48.4)	247 (48.1)	47 (50.0)	0.822 ^a^	216 (47.4)	74 (5.1)	0.093 ^a^
NEWS2 *	6 [4–8]	5 [4–8]	7 [5–9]	<0.001 ^b^	5 [4–7]	4.5 [4–6]	0.870 ^b^
CCI *	4 [2–5]	4 [2–5]	4 [2–5]	0.087 ^b^	3 [2–5]	5 [4–7]	<0.001 ^b^
SaO2 > 90% (N, %)	459 (75.7)	422 (82.2)	38 (40.4)	<0.001 ^a^	364 (80.0)	94 (71.2)	0.042
#SaO2 > 90% (N, %)	599 (98.3)	433 (84.4)	28 (28.9)	0.012 ^a^	411 (90.1)	50 (37.9)	0.070
HR (bpm) *	81 [74–94]	85 [75–100]	88 [80–100]	0.016 ^b^	84 [75–97]	84 [68–96]	0.015 ^b^
SBP (mmHg) *	130 [120–144]	131 [120–150]	130 [116–148]	0.503 ^b^	130 [120–146]	122 [106–140]	0.015 ^b^
Hb (g/dL) *	13.1 [11.9–14.3]	13.0 [11.3- 14.1]	13.1 [11.4–14.3]	0.689 ^b^	13.2 [12.0–14.5]	12.5 [11.9–14.3]	0.773 ^b^
WBCs (*10^3^/mmc) *	7.4 [6.0–10.3]	7.7 [5.5–10.7]	10.0 [7.0–14.3]	<0.001 ^b^	7.3 [5.8–10.1]	11.1 [7.3–14.5]	<0.001 ^b^
#WBCs (*10^3^/mmc) *	9.4 [6.6–13.1]	9.0 [6.6–12.2]	12.5 [8.7–17.7]	<0.001 ^b^	9.6 [6.5–12.0]	11.1 [8.8–12.7]	0.003 ^b^
Ly (*10^3^/mmc) *	1.1 [0.7–1.6]	1.1 [0.8–1.6]	0.9 [0.5–1.4]	0.001 ^b^	1.3 [1.0–1.7]	1.0 [0.7–1.8]	0.923 ^b^
#Ly (*10^3^/mmc) *	1.3 [0.9–1.9]	1.5 [1.0–2.0]	1.0 [0.5–1.5]	<0.001 ^b^	1.6 [1.2–2.1]	0.9 [0.7–2.1]	<0.001 ^b^
RDW (%) *	13.8 [13.1–15.4]	13.8 [13.0–15.0]	14.4 [13.4–15.9]	0.242 ^b^	13.6 [12.9–15.1]	15.3 [13.8–15.9]	<0.001 ^b^
MPV *	10.7 [9.9–11.3]	10.6 [10.0–11.3]	10.8 [10.3–11.7]	0.176 ^b^	10.8 [9.8–11.4]	10.7 [10.0–11.9]	0.052 ^b^
MPR *	0.3 [0.2–0.4]	0.3 [0.2–0.4]	0.2 [0.1–0.4]	0.191 ^b^	0.3 [0.2–0.4]	0.4 [0.3–0.5]	0.002 ^b^
NLR *	4.6 [2.8–7.2]	5.2 [3.0–9.3]	9.6 [4.8–18.6]	<0.001 ^b^	4.4 [2.8–6.7]	7.1 [5.4–10.2]	0.078 ^b^
PLR *	181.5 [126.6–304.8]	208.8 [137.9–333.1]	267.9 [155.9–452.8]	0.004 ^b^	169.6 [125.9–306.5]	174.7 [131.1–305.6]	0.230 ^b^
MLR *	0.5 [0.4–0.8]	0.5 [0.4–0.8]	0.6 [0.4–1.1]	0.012 ^b^	0.5 [0.4–0.8]	0.9 [0.5–1.1]	0.066 ^b^
#NLR *	3.8 [2.5–6.4]	4.2 [2.5–7.4]	15.9 [7.3–30.5]	<0.001 ^b^	3.9 [2.6–5.2]	6.5 [2.8–13.1]	<0.001 ^b^
#PLR *	201.8 [129.2–294.2]	199.0 [135.0–292.2]	343.1 [160.4–530.3]	<0.001 ^b^	198.7 [128.5–283.8]	219.8 [134.2–292.2]	0.928 ^b^
#MLR *	0.4 [0.3–0.7]	0.4 [0.3–0.7]	0.7 [0.4–1.3]	<0.001 ^b^	0.4 [0.3–0.6]	0.9 [0.3–1.4]	<0.001 ^b^
SII *	1075.7 [501.5–1912.0]	1164.4 [585.1–2297.0]	2031.8 [930.0–4579.0]	<0.001 ^b^	1110.5 [465.4–1973.0]	1628.8 [862.0–2812.8]	0.642 ^b^
#SII *	1202.8 [712.1–2044.2]	1170.2 [654.7–2211.1]	3915.5 [1202.5–7348.2]	<0.001 ^b^	1347.0 [699.5–1826.2]	1689.9 [548.2–2689.6]	0.022 ^b^
NMR *	9.2 [6.5–13.6]	9.0 [6.5–13.1]	10.9 [7.6–16.4]	<0.001 ^b^	8.3 [6.4–11.8]	11.9 [8.5–13.4]	0.527 ^b^
NPR *	2.2 [1.7–3.3]	2.4 [1.7–3.7]	3.5 [2.5–5.1]	<0.001 ^b^	2.2 [1.7–3.2]	3.5 [1.9–5.6]	<0.001 ^b^
ESR (mm/h) *	44 [10–66]	36 [11–60]	51 [7–72]	0.416 ^b^	31 [11–65]	52 [17–74]	0.474 ^b^
PT (seconds) *	12.9 [12.0–14.6]	13.1 [12.0–15.0]	13.2 [12.1–15.0]	0.381 ^b^	12.7 [12.0–14.6]	12.9 [11.7–15.5]	0.288 ^b^
Fibrinogen (mg/dL) *	430 [358–488]	444 [363–533]	468 [381–573]	0.032 ^b^	417 [354–473]	425 [363–538]	0.202 ^b^
#Fibrinogen (mg/dL) *	381 [315–464]	381 [319–450]	366 [283–483]	0.212	381 [319–454]	373 [267–463]	0.045
Lactate (mg/dL) *	22.6 [14.1–29.4]	22.8 [14.4–28.5]	28.9 [18.7–35.4]	0.032 ^b^	26.0 [21.3–30.2]	27.8 [23.8–31.2]	0.212 ^b^
CRP (mg/dL) *	5.0 [1.5–9.9]	5.8 [1.9–13.4]	12.3 [4.4–20.4]	<0.001 ^b^	3.8 [1.7–8.0]	8.6 [1.5–10.5]	0.464 ^b^
#CRP (mg/dL) *	1.4 [0.4–5.4]	1.6 [0.4–5.3]	4.2 [1.4–8.4]	<0.001 ^b^	0.9 [0.3–3.7]	6.4 [1.1–9.4]	<0.001 ^b^
CLR	6.0 [1.6–17.3]	3.9 [1.1–11.6]	8.1 [2.4–23.2]	<0.001 ^b^	3.5 [1.2–7.0]	5.4 [2.0–11.1]	0.565 ^b^
#CLR	1.4 [0.3–5.2]	1.2 [0.3–4.1]	6.2 [1.2–14.6]	<0.001 ^b^	0.4 [0.1–3.2]	6.0 [0.5–15.1]	<0.001 ^b^
Creatinine (mg/dL) *	0.89 [0.73–1.19]	0.89 [0.76–1.20]	1.04 [0.81–1.49]	<0.001 ^b^	0.8 [0.7–1.2]	1.3 [0.9–2.3]	<0.001 ^b^
TGP (U/L) *	31 [18–52]	32 [20–52]	38 [23–58]	0.079 ^b^	28 [19–47]	38 [31–63]	0.415 ^b^
LDH (U/L) *	240 [200–314]	266 [204–396]	489 [317–685]	<0.001 ^b^	242 [200–295]	219 [193–323]	0.124 ^b^
#LDH (U/L) *	197 [168–254]	202 [163–272]	463 [255–628]	<0.001 ^b^	200 [160–246]	330 [186–442]	<0.001 ^b^
Presepsin (ng/mL) *	331 [192–597]	336 [186–577]	727 [387–1844]	<0.001 ^b^	306 [156–433]	667 [407–1117]	<0.001 ^b^
Ferritin (ng/dL) *	449 [192–881]	495 [195–1059]	757 [376–1519]	<0.001 ^b^	295 [174–783]	448 [212–799]	0.980 ^b^
Hospitalization (days)	15 [12–18]	15 [12–19]	14 [10–18]	0.133 ^b^	15 [13–18]	16 [14–19]	0.001
Duration of illness (days)	19 [16–22]	15 [8–19]	14 [9–20]	0.566 ^b^	19 [17–23]	18 [15–20]	<0.001 ^b^

%, percentage of total cases within category; ^a^, using Chi-squared; *, data are presented as the median [25–75]; ^b^, using Mann–Whitney test; SaO2, oxygen saturation; NEWS2, National Early Warning Score 2; CCI, Charlson comorbidity index; Hb, hemoglobin; bpm; beats/minute; SBP, systolic blood pressure; WBC, white blood cell; Ly, lymphocyte count; RDW, red cell distribution width; MPV, mean platelet volume; NLR, neutrophil-to-lymphocyte ratio; PLR, platelet-to-lymphocyte ratio; MLR, monocyte-to-lymphocyte ratio; #, value obtained on follow up; SII, systemic immune-inflammation index; NMR, neutrophil-to-monocyte ratio; NPR, neutrophil-to-platelet ratio; ESR, erythrocyte sedimentation rate; PT, prothrombin time; CRP, C-reactive protein; CLR, C-reactive protein-to-lymphocyte ratio; TGP, alanine aminotransferase; LDH, lactate dehydrogenase.

**Table 2:** Logistic regression models for the main outcomes’ prediction including follow-up NLR.

Variable	ICU Admission				Short-Term Mortality			
	Univariate		Multivariate		Univariate		Multivariate	
	OR (95%CI)	*p* Value	OR (95%CI)	*p* Value	OR (95%CI)	*p* Value	OR (95%CI)	*p* Value
NLR #	1.08 (1.05–1.11)	<0.001	1.14 (1.06–1.22)	<0.001	1.15 (1.11–1.19)	<0.001	1.30 (1.09–1.57)	0.005
Deltastrain	1.50 (1.23–1.83)	<0.001	2.34 (1.12–4.90)	0.024	-	-	-	-
SaO2 (<90%)	1.15 (1.09–1.24)	<0.001	2.74 (0.70–10.73)	0.149	2.55 (1.89–3.42)	<0.001	1.80 (0.30–10.90)	0.520
NEWS2	1.20 (1.11–1.28)	<0.001	1.06 (0.84–1.36)	0.616	-	-	-	-
CCI	-	-	-	-	1.35 (1.24–1.46)	<0.001	1.49 (1.04–2.13)	0.030
SII	1.00 (1.00–1.00)	<0.001	1.00 (1.00–1.00)	0.005	-	-	-	-
SII #	-	-	-	-	1.00 (1.00–1.00)	<0.001	1.00 (1.00–1.00)	0.056
Fibrinogen	1.00 (1.00–1.00)	0.012	1.00 (1.00–1.01)	0.151	-	-	-	-
Fibrinogen #	-	-	-	-	1.00 (1.00–1.00)	0.056	1.00 (0.99–1.01)	0.962
CRP #	1.07 (1.03–1.10)	<0.001	0.91 (0.79–1.04)	0.165	1.14 (1.09–1.19)	<0.001	1.04 (0.92–1.19)	0.517
LDH #	1.00 (1.00–1.00)	<0.001	1.00 (1.00–1.01)	0.127	1.01 (1.01–1.01)	<0.001	1.01 (1.00–1.01)	0.013
Presepsin	0.02 (1.00–1.00)	<0.001	1.00 (1.00–1.00)	<0.001	1.00 (1.00–1.00)	<0.001	1.00 (1.00–1.00)	0.004
OD	1.31 (0.70–2.45)	0.040	0.82 (0.11–6.45)	0.851	1.45 (0.93–2.24)	0.098	2.50 (0.47–13.20)	0.281
Duration of illness	-	-	-	-	0.94 (0.92–0.96)	<0.001	1.14 (1.02–1.26)	0.017

NLR, neutrophil-to-lymphocyte ratio; #, value obtained on follow up; -, variable was not used in the model; SaO2, oxygen saturation; NEWS2, National Early Warning Score 2; CCI, Charlson comorbidity index; SII, systemic immune-inflammation index; CRP, C-reactive protein; LDH, lactate dehydrogenase; OD, oncologic disease.

**Table 3:** Logistic regression models for the main outcomes’ prediction including follow-up MLR.

Variable	ICU Admission				Short-Term Mortality			
	Univariate		Multivariate		Univariate		Multivariate	
	OR (95%CI)	*p* Value	OR (95%CI)	*p* Value	OR (95%CI)	*p* Value	OR (95%CI)	*p* Value
MLR #	1.08 (1.05–1.11)	<0.001	2.46 (1.27–4.79)	0.008	2.05 (1.43–2.93)	<0.001	2.23 (1.07–4.65)	0.032
Delta strain	1.50 (1.23–1.83)	<0.001	2.48 (1.25–4.92)	0.010	-	-	-	-
SaO2 (<90%)	1.15 (1.09–1.24)	<0.001	5.62 (1.51–20.91)	0.010	2.55 (1.89–3.42)	<0.001	1.58 (0.31–8.07)	0.586
NEWS2	1.20 (1.11–1.28)	<0.001	1.07 (0.85–1.35)	0.549	-	-	-	-
CCI	-	-	-	-	1.35 (1.24–1.46)	<0.001	1.47 (1.05–2.05)	0.024
SII	1.00 (1.00–1.00)	<0.001	1.00 (1.00–1.00)	0.004	-	-	-	-
SII #					1.00 (1.00–1.00)	<0.001	1.00 (1.00–1.00)	0.183
Fibrinogen	1.00 (1.00–1.00)	0.012	1.00 (1.00–1.01)	0.173	-	-	-	-
Fibrinogen #	-	-	-	-	1.00 (1.00–1.00)	0.056	1.00 (0.99–1.01)	0.940
CRP #	1.07 (1.03–1.10)	<0.001	0.92 (0.80–1.06)	0.229	1.14 (1.09–1.19)	<0.001	1.05 (0.93–1.19)	0.404
LDH #	1.00 (1.00–1.00)	<0.001	1.00 (1.00–1.00)	0.057	1.01 (1.01–1.01)	<0.001	1.01 (1.00–1.01)	0.002
Presepsin	0.02 (1.00–1.00)	<0.001	1.00 (1.00–1.00)	<0.001	1.00 (1.00–1.00)	<0.001	1.00 (1.00–1.00)	0.002
OD	1.31 (0.70–2.45)	0.040	1.41 (0.19–10.28)	0.735	1.45 (0.93–2.24)	0.098	2.21 (0.45–11.00)	0.331
Duration of illness	-	-	-	-	0.94 (0.92–0.96)	<0.001	1.11 (1.01–1.22)	0.036

MLR, monocyte-to-lymphocyte ratio; #, value obtained on follow up; -, variable was not used in the model; SaO2, oxygen saturation; NEWS2, National Early Warning Score 2; CCI, Charlson comorbidity index; SII, systemic immune-inflammation index; CRP, C-reactive protein; LDH, lactate dehydrogenase; OD, oncologic disease.

**Table 4:** Logistic regression models testing NLR, MLR and other inflammatory biomarkers in relation to the main outcomes.

Variable	ICU Admission			Short-Term Mortality		
	OR (95%CI) Model 1	OR (95%CI) Model 2	OR (95%CI) Model 3	OR (95%CI) Model 1	OR (95%CI) Model 2	OR (95%CI) Model 3
NLR #	1.14 (1.06–1.22)		1.12 (1.04–1.20)	1.30 (1.09–1.57)		1.28 (1.06–1.54)
MLR #	-	2.46 (1.27–4.79)	1.92 (0.79–4.71) *		2.23 (1.07–4.65)	1.84 (0.68–4.95) *
Delta strain	2.34 (1.12–4.90)	2.48 (1.25–4.92)	2.37 (1.13–4.97)			
SaO2 (<90%)	-	5.62 (1.51–20.91)	1.29 (1.21–1.70) *	-	-	-
CCI				1.49 (1.04–2.13)	1.47 (1.05–2.05)	1.50 (1.04–2.16)
SII	1.00 (1.00–1.00)	1.00 (1.00–1.00)	1.00 (1.00–1.00)			
SII #				1.00 (1.00–1.00) *	-	1.00 (1.00–1.00) *
Fibrinogen	-	-	1.01 (1.00–1.01) *			
Fibrinogen #				-	-	-
CRP #	-	-	-	-	-	-
LDH #	-	1.00 (1.00–1.00) *	-	1.01 (1.00–1.01)	1.01 (1.00–1.01)	1.01 (1.00–1.01)
Presepsin	1.00 (1.00–1.00)	1.00 (1.00–1.00)	1.00 (1.00–1.00)	1.00 (1.00–1.00)	1.00 (1.00–1.00)	1.00 (1.00–1.00)
OD	-	-	-	-	-	-
Duration of illness				1.30 (1.09–1.57)	1.11 (1.01–1.22)	1.14 (1.02–1.26)
ROC analysis	AUC (95%CI)	AUC (95%CI)	AUC (95%CI)	AUC (95%CI)	AUC (95%CI)	AUC (95%CI)
	0.937 (0.880–0.994)	0.912 (0.838–0.986)	0.939 (0.882–0.997)	0.948 (0.911–0.984)	0.931 (0.888–0.975)	0.950 (0.914–0.986)

#, value obtained on follow up; -, variables were not statistically significant in the model; *, *p* < 0.1.

## Data Availability

The data presented in this study are available in this article and the Supplementary Material.

## Supplementary Materials

The following supporting information can be downloaded at: https://www.mdpi.com/article/10.3390/jpm12122037/s1, Table S1: Logistic regression for models including both follow-up NLR and MLR in relation to the main outcomes.
