# Supplementary material for: The Value of Early and Follow-Up Elevated Scores Based on Peripheral Complete Blood Cell Count for Predicting Adverse Outcomes in COVID-19 Patients
Source: J Pers Med. 2022 Dec 9;12(12):2037. doi: 10.3390/jpm12122037 (PMC9781715; doi:10.3390/jpm12122037)
Supplement: Supplementary file 1 [file jpm-12-02037-s001.zip › jpm-2020089-Supplementary.pdf]

**Table S1.** Logistic regression for models including both follow-up NLR and MLR in relation with the main outcomes.

| Variable | ICU admission | | | | Short-term mortality | | | |
|---|---|---|---|---|---|---|---|---|
| | Univariate | | Multivariate | | Univariate | | Multivariate | |
| | OR (95%CI) | P value | OR (95%CI) | P value | OR (95%CI) | P value | OR (95%CI) | P value |
| **NLR#** | 1.10 (1.07-1.12) | <0.001 | 1.12 (1.04-1.20) | 0.003 | 1.15 (1.11-1.19) | <0.001 | 1.28 (1.06-1.54) | 0.009 |
| **MLR#** | 1.08 (1.05-1.11) | <0.001 | 1.92 (0.79-4.71) | 0.152 | | | 1.84 (0.68-4.95) | 0.092 |
| **Viral strain (Delta)** | 1.50 (1.23-1.83) | <0.001 | 2.37 (1.13-4.97) | 0.023 | - | - | - | - |
| **SaO2 (<90%)** | 1.15 (1.09-1.24) | <0.001 | 1.29 (1.21-1.70) | 0.090 | 2.55 (1.89-3.42) | < 0.001 | 1.66 (0.27-10.31) | 0.588 |
| **NEWS2 Score** | 1.20 (1.11 – 1.28) | <0.001 | 1.09 (0.86–1.40) | 0.464 | - | - | - | - |
| **CCI** | - | - | - | - | 1.35 (1.24-1.46) | < 0.001 | 1.50 (1.04-2.16) | 0.031 |
| **SII** | 1.00 (1.00-1.00) | <0.001 | 1.00 (1.00-1.00) | 0.014 | - | - | - | - |
| **SII#** | - | - | - | - | 1.00 (1.00-1.00) | < 0.001 | 1.00 (1.00-1.00) | 0.078 |
| **Fibrinogen** | 1.00 (1.00-1.00) | 0.012 | 1.01 (1.00-1.01) | 0.094 | - | - | - | - |
| **Fibrinogen#** | - | - | - | - | 1.00 (1.00-1.00) | 0.056 | 1.00 (0.99-1.01) | 0.950 |
| **CRP#** | 1.07 (1.03-1.10) | <0.001 | 0.91 (0.79-1.01) | 0.165 | 1.14 (1.09-1.19) | < 0.001 | 1.04 (0.91-1.19) | 0.544 |
| **LDH#** | 1.00 (1.00-1.00) | <0.001 | 1.00 (1.00-1.01) | 0.111 | 1.01 (1.01-1.01) | < 0.001 | 1.01 (1.00-1.01) | 0.011 |

| | | | | | | | | |
|---|---|---|---|---|---|---|---|---|
| **Presepsin** | 0.02 (1.00-1.00) | <0.001 | 1.00 (1.00-1.00) | <0.001 | 1.00 (1.00-1.00) | < 0.001 | 1.00 (1.00-1.00) | 0.005 |
| **OD** | 1.31 (0.70-2.45) | 0.040 | 1.41 (0.19-10.28) | 0.735 | 1.45 (0.93-2.24) | 0.098 | 2.65 (0.49-14.43) | 0.259 |
| **Duration of illness** | - | - | - | - | 0.94 (0.92-0.96) | < 0.001 | 1.14 (1.02-1.26) | 0.018 |

#, value obtained on follow-up; -, variable not used in the model; SaO2, oxygen saturation
